# Pre-validation methods for developing a patient reported outcome instrument

**DOI:** 10.1186/1471-2288-11-112

**Published:** 2011-08-09

**Authors:** Maria E Prior, Jemaima Che Hamzah, Jillian J Francis, Craig R Ramsay, Mayret M Castillo, Susan E Campbell, Augusto Azuara-Blanco, Jennifer M Burr

**Affiliations:** 1 Health Services Research Unit, University of Aberdeen, Aberdeen, AB25 2ZD, UK; 2 Department of Ophthalmology, Faculty of Medicine, Universiti Kebangsaan Malaysia, Kuala Lumpur, Malaysia; 3 Department of Ophthalmology, Aberdeen Royal Infirmary, Aberdeen, UK; 4 School of Nursing and Midwifery, University of East Anglia, Norwich, UK

## Abstract

**Background:**

Measures that reflect patients' assessment of their health are of increasing importance as outcome measures in randomised controlled trials. The methodological approach used in the pre-validation development of new instruments (item generation, item reduction and question formatting) should be robust and transparent. The totality of the content of existing PRO instruments for a specific condition provides a valuable resource (pool of items) that can be utilised to develop new instruments. Such 'top down' approaches are common, but the explicit pre-validation methods are often poorly reported. This paper presents a systematic and generalisable 5-step pre-validation PRO instrument methodology.

**Methods:**

The method is illustrated using the example of the Aberdeen Glaucoma Questionnaire (AGQ). The five steps are: 1) Generation of a pool of items; 2) Item de-duplication (three phases); 3) Item reduction (two phases); 4) Assessment of the remaining items' content coverage against a pre-existing theoretical framework appropriate to the objectives of the instrument and the target population (e.g. ICF); and 5) qualitative exploration of the target populations' views of the new instrument and the items it contains.

**Results:**

The AGQ 'item pool' contained 725 items. Three de-duplication phases resulted in reduction of 91, 225 and 48 items respectively. The item reduction phases discarded 70 items and 208 items respectively. The draft AGQ contained 83 items with good content coverage. The qualitative exploration ('think aloud' study) resulted in removal of a further 15 items and refinement to the wording of others. The resultant draft AGQ contained 68 items.

**Conclusions:**

This study presents a novel methodology for developing a PRO instrument, based on three sources: literature reporting what is important to patient; theoretically coherent framework; and patients' experience of completing the instrument. By systematically accounting for all items dropped after the item generation phase, our method ensures that the AGQ is developed in a transparent, replicable manner and is fit for validation. We recommend this method to enhance the likelihood that new PRO instruments will be appropriate to the research context in which they are used, acceptable to research participants and likely to generate valid data.

## Background

Measures that reflect patients' assessment of their health are of increasing importance as outcome measures in randomised controlled trials (RCTs). The decision whether to use a validated patient reported outcome (PRO) instrument or to develop a new one should be based on a thorough review of PRO instruments used in a population of interest [1]. If a new PRO instrument is required, the methodological approach used in its development should be robust and transparent. There are two main phases in generating a new PRO instrument - developing the instrument and validating the instrument. Steps for developing a PRO instrument involve item generation, item reduction and question formatting. Validation of the instrument follows, to assess coherence across the items and inform the removal of poorly discriminating, unreliable or invalid items [2].

To generate a pool of potentially relevant items for condition-specific instruments, most studies focus on an inductive 'bottom up' approach using qualitative methods (e.g. focus groups or one-to-one interviews with the target population), which ensures items reflect the perspective of the majority of individuals in the population of interest [3,4]. However, data generated using this approach often reaffirm previous qualitative findings, resulting in the development of 'new' instruments containing items with overlapping, but not identical content coverage [3-5].

As the catalogue of validated PRO instruments grows within a clinical specialty, so does the body of empirical evidence of what is important to patients with that condition ('content' domains). The body of evidence represented by the totality of the content of existing PRO instruments for a specific condition may provide a valuable resource (pool of items) that can be utilised to develop new PRO instruments. Such 'top down' approaches, using expert opinion and/or the published literature in the field, are common [6] but the explicit methods used in the item generation and item reduction stages are often poorly reported [7,8].

To address the lack of explicit methods for item generation and reduction, this paper presents a 5-step methodology for the pre-validation stages of PRO instrument development (i.e. item generation, item reduction and question formatting). The method is illustrated using the example of the Aberdeen Glaucoma Questionnaire (AGQ), a new instrument designed to be used for a future randomised controlled trial (RCT) evaluating the effectiveness of glaucoma screening compared with no formal screening (opportunistic case detection)

## Methods

### Example instrument - Aberdeen Glaucoma Questionnaire

The aim of the AGQ is to compare patient reported vision related disability between the intervention (glaucoma screening) and comparator (opportunistic case detection) arms at the end of a proposed RCT. In ophthalmology there are high quality validated vision and glaucoma PRO instruments covering a wide range of content (e.g. visual impairment, visual symptoms, treatment satisfaction, activity and participation difficulties) [9]. However, existing instruments used in glaucoma populations are not suitable in their entirety for evaluating screening interventions because the trial population includes people with and without a diagnosis [9]. In addition, within a screening trial context it is also important to compare other health effects between the screened and unscreened population to capture any wider benefits or harms of screening. Existing generic instruments are adequate for this purpose and can be used alongside condition-specific measures. Given this context, we proposed to build on previous research by making use of the existing body of knowledge in terms of items that are known to be relevant to people with glaucoma and to develop a new instrument from these.

Table 1 presents the five steps involved in the pre-validation PRO development methodology. Steps 1 to 3 involve the synthesis of the products of research (i.e. validated PRO instruments). The items that result from the systematic application of these steps form the basis of a new condition-specific PRO instrument. Step 4 involves assessment of the content coverage of items retained after Step 3 against a pre-existing theoretical framework appropriate to the objectives of the instrument and the target population. This process provides clarity on the dimensions of health covered in the new PRO instrument (i.e. how well the construct under measurement is represented by an instrument). Step 5 comprises a qualitative exploration of the target populations' views of the new instrument and the items it contains.

**Table 1:** 5-step PRO development methodology

Step 1	Item generation: a. Systematic identification of existing PRO instruments meeting explicit eligibility criteria. b. Selection of additional instruments (e.g. generic instruments) to be administered alongside the new PRO instrument. c. All items from the identified instruments form the initial 'item pool' (to which Steps 2-5 are applied).
Step 2	Item de-duplication. Items are discarded if: A) They are literal duplications (identically worded items, or duplication of item content) B) Their content differs only by timeframe or attribution to a condition of interest (e.g. *do you have difficulty... because of your condition*) C) Their content overlaps with generic measures to be administered alongside new instrument (e.g. SF-36)

Step 3	Item reduction: D) Macro level: items discarded if associated with content themes (dimensions of health) that are not appropriate for inclusion in the new instrument (e.g. treatment satisfaction) E) Micro level: application of explicit, study-relevant criteria to select items for inclusion in draft instrument (actual content area)

Step 4	Assessment of content coverage against a relevant pre-existing theoretical framework (revisit 3E if content coverage suboptimal)

Step 5	Exploratory pilot work with target population to assess comprehensibility, acceptability, relevance and answerability in order to inform instrument refinement (item removal &/or re-wording) (e.g.'think aloud' study, focus groups)

#### 1. Item generation for the AGQ

a) We selected instruments from PRO instruments used in a glaucoma context (generic, vision- and glaucoma-specific) that had been systematically identified as part of a wider study assessing instrument quality [9] using the following eligibility criteria: suitable for self report; validated in a glaucoma population; in the public domain; items and response options fully described in the text article reporting the instrument. The total content of the selected instruments provided a comprehensive pool of items, relevant to people with glaucoma, from which to select the 'best' combination of items to meet the specific scope of a new instrument.

b) The widely validated generic measure SF-36 was added to the list of selected instruments as it would be administered alongside the glaucoma-specific AGQ, in the proposed trial.

c) A database consisting of all items from the selected instruments (item pool) was created with items grouped according to content (e.g. reading, driving, walking). All information relating to the instrument of origin, the item and response option content and wording was retained.

#### 2. Item de-duplication for the AGQ

We conducted three phases of de-duplication (A, B, C) in the development of the draft AGQ.

A) In the first de-duplication task, three pairs of reviewers from the multi-disciplinary research team (health services researchers, health psychologists and ophthalmologists) independently assessed one third of all items for literal duplications. Literal duplication was defined as identically worded items (including timeframe), or items which, in each reviewer's opinion, asked the same question (but may be worded differently) and which, if both were included in the AGQ, would represent duplication of content. One researcher (MP) collated decisions from each pair of reviewers. Items for which there was reviewer agreement were reduced to a single item in the item pool, with all other information retained (i.e. instrument of origin, response options and unique item number). Any reviewer disagreements were resolved by consensus or arbitration by JB (clinical academic (ophthalmology)).

B) The second phase of item de-duplication involved grouping together all items that referred to the same aspect of a specific content theme (e.g. loneliness), but which differed in timeframe or in whether they included an attribution of action to eyesight (e.g. *do you feel alone? *versus *in the past month have you felt lonely or isolated because of your eyesight?*). Such items were grouped together and the wording of all individual items within each group was retained. The purpose of this phase of de-duplication was to retain all aspects of all content themes within the item pool, whilst acknowledging that only one item per group would ultimately be chosen for inclusion in the AGQ.

C) The third phase of de-duplication identified item content overlap between the SF-36 items and other items in the pool. All SF-36 items and any extra items considered, by the multi-disciplinary team, to be directly covered by SF-36 items were removed from the item pool.

#### 3. Item reduction for the AGQ

Two phases of item reduction (D and E) were conducted to determine which items were retained in the draft AGQ.

D) The first phase of item reduction consisted of a 'macro level' removal of items relating to content themes that were not appropriate for inclusion in the AGQ; a self-report measure of vision related disability. Decisions regarding the removal of all items within a content theme (e.g. treatment satisfaction) were informed by the literature and by the multi-disciplinary research team including the clinical opinion of the three ophthalmologists involved in the project.

E) All items remaining after Phase D covered content themes (e.g. driving, reading, using public transport) relevant to vision related disability associated with glaucoma. However, the specific wording of many of these items made them unsuitable for inclusion in the draft AGQ. Explicit criteria were applied to identify items for removal. All items that were not applicable to people without a diagnosis of glaucoma were removed. As were items referring to 'frequency of' rather than 'difficulty with' performing an activity (e.g. driving). Items relating to very specific tasks (e.g. difficulty threading a needle) were removed to reduce participant burden and to accommodate the inclusion of similar, but more widely applicable items (e.g. difficulty with tasks that require you to see up close). Item quality was assessed by the multi-disciplinary team. Items with poor reading ease and/or potential ambiguity were removed (e.g. *"Do you hit persons or objects?")*. Following phase E of the item reduction, the wording of the remaining items was adapted to maximise consistency of both item wording and response format. Items were then formatted as the draft AGQ.

#### 4. Assessing AGQ content coverage against a pre-existing theoretical framework

We used the World Health Organisation (WHO) International Classification of Functioning, Disability and Health (ICF) [10] to assess the content coverage of the AGQ. The ICF provides a framework within which item content can be systematically coded using a standardised common language [10] thus providing clarity on how well the construct under measurement (i.e. vision related disability associated with glaucoma) is represented within the AGQ. The ICF takes into account the social aspects, not just the medical or biological aspects of disability, and defines four components of functioning and disability: Body Structures, Body Functions, Activities and Participation [10]. In addition, the ICF contains contextual Environmental Factors (i.e. physical, attitudinal and social factors) and Personal Factors (e.g. age, gender) that might influence functioning and disability. The content of each included item in the draft AGQ was assessed for its coverage of ICF components and contextual factors using the linking rules developed by Cieza and colleagues (2005) [11]. This process involves first identifying the *meaningful concepts *(i.e. the ideas or information) contained within each item in the AGQ. These might relate to body structures (e.g. the eye) or functions (e.g. seeing) or to activities (e.g. walking) or to participation in a life situation (e.g. using public transport). Each meaningful concept is then linked to the most precise ICF category and coded accordingly (e.g. visual field = b2101, light intensity = e2400) [10].

#### 5. Exploratory pilot work - A 'think aloud' study

The 'think aloud' technique (cognitive interviewing) is a valuable method for gaining insights into people's thought processes whilst undertaking a task [12]. The technique is commonly used during questionnaire development to determine whether the meaning of an item, as intended by the questionnaire developer, is consistent with the respondent's interpretation of that item [13]. A 'think aloud' study was conducted to explore glaucoma patients' views on the comprehensibility, acceptability, relevance and answerability of the draft AGQ in order to inform its refinement. Ethical approval was obtained from the North of Scotland Research Ethics Committee (Ref: 09/S0801/41)

Participants consisted of a purposive sample of eight patients with differing severities of glaucoma (two each with mild, moderate or severe glaucoma and two with no visual impairment). Each participant completed the AGQ, in the presence of a researcher (MC), who asked them to verbalise their thoughts ('think aloud') whilst completing a paper version of the draft AGQ. In addition, MC probed patients' comments about specific items and ascertained their opinions about the AGQ in general (e.g. content, format, length). Interviews were audio-recorded and transcribed verbatim. Verbal responses to each item were tabulated alongside a participant's written response to that item to identify possible impact of item interpretation on written responses. We used an 'item centred' coding scheme based on Tourangeau's Cognitive Aspect of Survey Methodology Framework to identify 'problem' items (i.e. those with which participants had difficulty following instructions or problems with comprehensibility, acceptability, relevance or answerability) [14]. MC coded all transcripts and MP double coded two transcripts (25% of the data). Disagreements were resolved by consensus of the 'think aloud' research team (MC, MP, JF, JB). The 'think aloud' findings informed the refinement of the AGQ.

## Results

**Figure 1:**
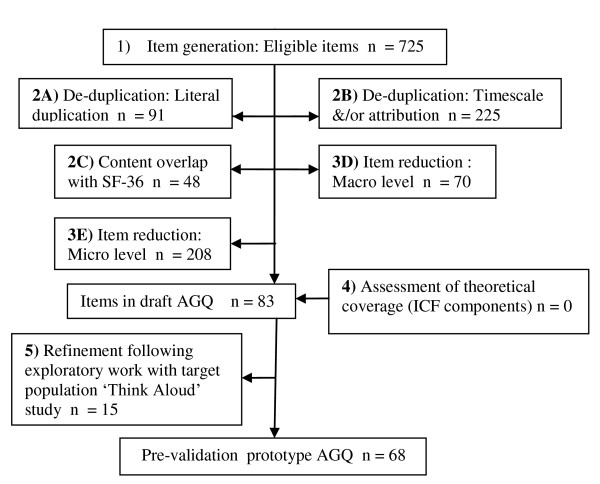
**methodological development of AGQ flowchart**.

Figure 1 presents an overview of the results in the development of the AGQ. Each of the steps represented in Figure 1 is described in detail below.

### 1. Item generation for the AGQ

The systematic review of PRO instruments used in the glaucoma populations identified 34 vision- or glaucoma-specific instruments and 7 generic instruments [9]. From these, we selected the 20 instruments (17 vision-specific, 3 generic) that met the selection criteria for inclusion in the item pool [15-34] (see Table 2). In addition, we included the SF-36 [35] in the selected instruments as it was to be an outcome measure in the proposed screening trial and including it allowed us to use these systematic methods to avoid duplication of content coverage between the SF-36 and items from other instruments.

**Table 2:** Selected instruments for inclusion in the item pool

Activities of daily vision scale [15]	TSS-IOP [16]
Glaucoma Symptom Scale [17]	Turano [18]

Glaucoma Quality of Life - 15 [19]	Uenishi [20]

IND VFQ 33 [21]	Visual Activities Questionnaire [22]

Impact of Vision Impairment [23]	VF-14 [24]

Mills [25]	LVQOL [26]

Viswanathan [27]	Adapted General Well-Being Index [28]

NEI-VFQ 25 [29]	CES-D [30]

Odberg symptom items [31]	SWED-QUAL [32]

QOLVFQ [33]	SUMI [34]

The deconstruction of eligible instruments and the SF-36 into constituent items generated an item pool of 725 items (Figure 1). When considered together, the total content of these 20 instruments provides a large assortment of items relevant to people with glaucoma. An extract from the item pool is shown in Table 3.

**Table 3:** Extract from item pool illustrating presentation of item-level data

Item No	Item	Response	Instrument
257	Do objects ever suddenly appear when you should have noticed them before?	1 = no, 2 = uncertain, 3 = yes	MILLS 1986

258	Does your vision give you any difficulty (even with glasses) with seeing objects coming from the side?	1 = none, 2 = a little bit, 3 = some, 4 = quite a lot, 5 = severe, 0 = do not perform for nonvisual reasons	GQL-15

259	Because of your eyesight, how much difficulty do you have noticing objects off to the side while you are walking along?	1 = no difficulty at all, 2 = a little difficulty, 3 = moderate difficulty, 4 = extreme difficulty, 5 = stopped doing this because of your eyesight, 6 = stopped doing this for other reasons or not interested in doing this	NEI VFQ-25

260	I have trouble noticing things in my peripheral vision.	never, rarely, sometimes, often, always	VAQ

### 2. Item de-duplication for the AGQ

#### Phase A

The literal duplication phase resulted in the reduction of 91 items. For example, Table 4 shows that four instruments include the item "*In general would you say your [overall] health is..."*, although response options vary. The stems of these four items were reduced to a single item, creating a reduction of three items from the item total (Table 5). All other information about items 2, 3, 4 and 5 was retained (i.e. instrument of origin, response option, unique item identification).

**Table 4:** Extract from item pool illustrating presentation of items 2, 3, 4, 5, *before *literal de-duplication

Item No.	Item	Response	Instrument
2	In general would you say your health is..	1-4 scale response, higher scores indicating more optimistic views.	QOLVFQ

3	In general, would you say your overall health is...	1 = Excellent, 2 = very good, 3 = good, 4 = fair, 5 = poor.	NEI VFQ-25

4	In general would you say your health is:	1 = very good, 2 = fairly good, 3 = fair, 4 = rather bad, 5 = very bad	SWED-QUAL

5	In general would you say your health is:	Excellent, very good, good, fair, poor	SF-36

**Table 5:** Extract from item pool illustrating presentation of items 2, 3, 4, 5, *after *literal de-duplication (phase A)

Item No.	Item	Response	Instrument
2	In general would you say your [overall] health is:	1-4 scale response, higher scores indicating more optimistic views.	QOLVFQ
		
3		1 = Excellent, 2 = very good, 3 = good, 4 = fair, 5 = poor.	NEI VFQ-25
		
4		1 = very good, 2 = fairly good, 3 = fair, 4 = rather bad, 5 = very bad	SWED-QUAL
		
5		Excellent, very good, good, fair, poor	SF-36

#### Phase B

The grouping of items that refer to the same aspect of a specific content theme, but which differ in timescale or attribution of action to eyesight resulted in the reduction of 225 items. The wording of all such items was retained, but row borders between them were removed. For example, two of the three items in Table 6 include a timescale (153, 68) and Item 68 asks participants to attribute any loneliness to their eyesight, whilst the other two do not.

**Table 6:** Phase B - Example of de-duplication on basis of differing timescale and/or attribution to eyesight

Item No.	Item	Response	Instrument
153	*During the past week *I felt lonely.	0 = rarely or none of the time (less than 1 day); 1 = some or a little of the time (1-2 days); 2 = occasionally or a moderate amount of the time (3-4 days); 3 = most or all of the time (5-7 days)	CES-D
		
154	Do you feel alone?	0 = no, 2 = sometimes, 4 = yes	UENISHI (2003)
		
68	*In the past month *have you felt lonely or isolated *because of your eyesight*?	not at all, very rarely, a little of the time, a fair amount of the time, a lot of the time, all of the time.	IVI

#### Phase C

The third phase of item de-duplication resulted in the reduction of 48 items (Figure 1); the 36 items from the SF-36 and 12 extra items considered to be directly covered by SF-36 items were removed. For instance, whilst not a literal duplication or only differing in timescale and/or attribution, the SF-36 item on bathing and dressing was considered to directly cover three items on dressing from two other instruments (Table 7).

**Table 7:** Extract from item pool after Phase C

Item No.	Item	Response	Instrument
594	During a typical day does your health limit bathing or dressing yourself?	Yes, limited a lot; yes, limited a little; No, not limited at al.	SF-36
		
597	Is your health today good enough that you can dress yourself?	1 = yes without difficult, 2 = yes, with some difficulty, 3 = yes, with great difficulty, 4 = no, not at all.	SWED-QUAL
		
598	Can you change clothes by yourself?	Yes/with difficulty/no	SUMI
		
601	Do you have difficulty dressing because of your visual problems?	No/occasionally/frequently	SUMI

### 3. Item reduction for the AGQ

#### Phase D

Decisions on which content themes were not relevant for inclusion in the AGQ were informed by the literature and by ophthalmologists on the research team. Excluded content themes include nausea, hearing, sleep, personal financial circumstances, treatment satisfaction and access to health services. This macro level item reduction (D) resulted in the removal of 70 items.

#### Phase E

The micro level reduction reduced the remaining 292 items to 83 items. Table 8 shows four such items removed in phase E. Although the content coverage of these items is relevant for inclusion in the AGQ, they relate to the performance of very specific tasks and were removed to reduce participant burden and to accommodate the inclusion of other, more widely applicable, items.

**Table 8:** Four of the items removed during Phase E

Item No.	Item	Response
553	When you write sentences in vertical lines, does it lean to either direction?	No/occasionally/frequently

566	Because of your vision how much problem do you have in locking and unlocking the door?	Not at all, a little, quite a bit, a lot, cannot do because of my sight.

569	Do you have difficulty, even with glasses doing decorating?	(Yes/No/Not applicable) If yes, how much difficulty do you currently have? (A little = 1; A moderate amount = 2; A great deal = 3, Are you unable to do the activity = 4?)

572	Do you have difficulty with chopsticks?	No/occasionally/frequently

### 4. Linking AGQ content to the ICF

The 83 items in the initial version of the AGQ originated from 16 of the 20 instruments used to generate the item pool in Step 1a [15,18,19,21-27,29-34]. The items contained meaningful concepts related to two ICF categories of Body Structure, eight of Body Function, fifteen categories associated with Activity or Participation and seven categories of Environmental factors. (See additional file 1: Linking AGQ content to ICF)

Inevitably, with such a collection of 83 items, item timescales varied in the initial version of the AGQ, as did response formats and the use of first- and second-person personal pronouns. Fifty-three of the selected items were formatted as questions; the others as statements or symptom checklists. This eclectic mix of 83 items represented the 'best' combination of content coverage with appropriate theoretical coverage. Item stem and response option wording was adapted to increase consistency in the new instrument, but the content and theoretical coverage of each item accurately reflected the original (i.e. in terms of *meaningful concept*). It was this initial draft of the AGQ (with 83 items) that was subjected, in Step 5, to further investigation using the 'think aloud' technique.

### 5. Results of Think Aloud study

The think-aloud interviews resulted in the removal of fifteen items from the initial version of the AGQ. Seven of these were removed due to comprehensibility problems (i.e. the participant either reported difficulties with the meaning of words, or they answered the item in such a way that suggested the item was not understood, or they reported a lack of contextual information needed to answer the item accurately (e.g. lack of timeframe). Importantly, the ICF category coverage was not affected by the removal of these, or any other, items. Removal of a further four items resulted from participants' suggestions of item redundancy (i.e. items covering difficulty with driving in different contexts were removed (e.g. driving in the rain at night with oncoming headlights) in the presence of five other driving items). The final four items removed resulted from the condensing of two-part items into single items following difficulties experienced by participants with correctly following re-routing instructions.

In addition to item removal, the think aloud study findings informed changes to the wording of retained items to increase consistency of item wording and minimise participant burden. For example in the initial version of the AGQ, the word *difficulty *appeared in many of the questions (do you have difficulty with...?), as well as in the corresponding response options (no difficulty, a little difficulty, moderate difficulty, extreme difficulty). The word *difficulty *was removed from the response options of such items, thus changing the wording of such responses to: no; a little; moderate; extreme. This change occurred in response to suggestions that the repetition in wording between question and response option was unnecessary and irritating.

### Summary of results

The systematic identification of 20 self-report instruments used in a glaucoma population and in the public domain, together with the SF-36, generated a total of 725 items, with extensive content coverage, for inclusion in an item pool. The application of three phases of de-duplication and two phases of item reduction resulted in the removal of a total of 642 items. The remaining 83 items were assessed to have a good breadth of coverage of ICF components (body structures, body functions, activity, participation and environment) and formed the initial draft of the AGQ. Where necessary the wording and formatting of items was adapted to increase consistency, but the content and theoretical coverage of each item was maintained. The exploration of comprehensibility, acceptability, relevance and answerability of the initial draft of the AGQ in a 'think aloud' study resulted in the removal of 15 items and to the refinement of others. The resultant pre-validation draft AGQ contains 68 items.

## Discussion

This paper outlines a novel methodological approach that is applicable to situations where a systematic literature review has identified numerous high quality validated instruments (developed using mainly inductive methods), but where none of the identified instruments are appropriate to be used, in their current form, to address the research questions in a particular study. Our method employs a systematic 5-step approach: 1) Generation of a pool of items from an existing body of knowledge; 2) Item de-duplication; 3) Item reduction; 4) Assessment of content validity (against a relevant pre-existing theoretical framework); and 5) Exploratory pilot work to assess comprehensibility, acceptability, relevance and answerability to the target population. Whilst our 5-step approach is largely 'top down', it differs from other studies using expert opinion and published literature to develop PRO instruments in that we systematically utilise the body of empirical evidence amassed from studies using 'bottom up' (qualitative) approaches.

We illustrated our pre-validation method using the example of the AGQ; a new glaucoma-specific PRO instrument for use as the primary patient reported outcome measure for a RCT evaluating the effectiveness of glaucoma screening. We describe how a large number of items representing the totality of the content of existing PRO instruments for a specific condition can be reduced to those included in a draft PRO instrument with appropriate content and theoretical coverage, and ready for validation. The methods we describe are applicable to the development of other PRO instruments and more widely to instrument development in other disciplines.

This paper addresses a methodological gap in the pre-validation instrument development literature, where there is a tendency for authors to report, in some detail, the item generation phase that result from qualitative work [3,5,36]. However, there are few reports of how a large number of items generated in early phase work (using qualitative methods, expert opinion and/or published literature) are reduced to those that are included in the draft PRO instrument used in the validation process.

The aim of the AGQ is to measure vision related disability associated with glaucoma and its treatment in a population screening trial. The use of the ICF as a theoretical framework enabled us to identify which aspects of vision related disability are covered in the AGQ and the balance of that coverage (i.e. body structures, body functions, activity and participation). Currently, the social context of disability is under represented in PRO instruments (over emphasis on items that measure body structures and functions) [9]. By contrast, the AGQ provides good breadth of coverage of ICF components from both an individual (body structures, body functions and activities) and societal (participation) perspective. This is important, if PRO instruments are to serve the purpose of complementing clinical outcomes (rather than duplicating them from the patients' perspective).

This study used a subsample of PRO instruments identified in a wider study assessing instrument quality. One of the reasons for this was the necessity to construct our item pool from instruments in the public domain. This inevitably resulted in the exclusion of valid and reliable instruments with relevant content. This limitation is unlikely to have had a detrimental effect on the content coverage of our item pool due to the diversity of the instruments included in this study and to the breadth of content and theoretical coverage of their items.

We do not yet know the psychometric properties of the AGQ and which, if any, items will be dropped as a result of a formal validation and assessment of its psychometric properties. However, this is true of all pre-validation work and does not detract from the importance of reporting this systematic approach to pre-validation methodology. The content of the AGQ has been designed to be acceptable to all participants in a future RCT evaluating the effectiveness of glaucoma screening (i.e. applicable to people with and without a diagnosis of glaucoma). In addition, we anticipate that the optimal AGQ will be able to discriminate between different stages of glaucoma severity. By systematically accounting for all items dropped after the item generation phase, our method ensures that the AGQ is fit for validation. The discriminative capabilities (responsiveness) of the AGQ will be established following formal validation with a large patient sample and will be reported elsewhere.

## Conclusions

This study presents a novel methodology for developing a new PRO instrument, based on three sources: literature that reports what is important to patients (content coverage) and which provides a body of empirical evidence for item generation; theoretically coherent framework (theoretical coverage); and patients' experience of completing the instrument (acceptability). We recommend this to researchers as a transparent and replicable method that will enhance the likelihood that new PRO instruments will be appropriate to the research context in which they are used, acceptable to research participants and likely to generate valid data.

## List of abbreviations

RCT: Randomised controlled trial; PRO: Patient reported outcome; AGQ: Aberdeen Glaucoma Questionnaire; WHO: World Health Organisation; ICF: International Classification of Functioning Disability and Health.

## Competing interests

The authors declare that they have no competing interests.

## Authors' contributions

MP participated in the design of the study, coordinated and conducted the study and drafted the manuscript. JC conducted the systematic review from which the subsample of PRO instruments used in a glaucoma population was selected and helped in the linking of the AGQ items to the ICF and in the drafting of the manuscript. JF and CR participated in the design of the study and in its conduct and helped to draft the manuscript. MC conducted the 'think aloud' study and helped draft the manuscript. SC and AAB participated in the conduct of the study and helped draft the manuscript. MP, JB, JF, JC, CR and SC conducted the item de-duplication task. JB conceived the study, participated in its design and conduct and helped to draft the manuscript. All authors were involved in the item reduction phase and have read and approved the final manuscript.

## Pre-publication history

The pre-publication history for this paper can be accessed here:

http://www.biomedcentral.com/1471-2288/11/112/prepub

## Supplementary Material

Additional file 1 **Linking AGQ content to ICF**. Click here for file
