# Supplementary material for: Pre-validation methods for developing a patient reported outcome instrument
Source: BMC Med Res Methodol. 2011 Aug 9;11:112. doi: 10.1186/1471-2288-11-112 (PMC3225127; doi:10.1186/1471-2288-11-112)
Supplement: Additional file 1 — Linking AGQ content to ICF. [file 1471-2288-11-112-S1.PDF]

Linking AGQ content to the ICF

Body Structures	**ICF Categories**		**ICF codes**
The eye, ear and related structures	Structure around eye		s230
	Structure of eye ball		s220
Body Functions	**ICF Categories**	**Sub categories**	**ICF codes**
Mental function	Specific mental function (Emotional function)		b152
Sensory function and pain	Seeing function		b210
		visual field	b2101
		Light sensitivity	b21020
		Colour vision	b21021
	Sensation of pain	Pain in body part, head and neck	b2801
		Pain in head and neck	b28010
	Hearing and vestibular function	Sensations associated with hearing and vestibular function (Sensation of falling)	b2402
	Additional sensory functions	Taste function	b250
	Perceptual function	Visual perception	b1561
Functions of cardiovascular, haematological, immunological & respiratory systems	Functions of respiratory system	Respiratory functions (Respiratory rhythm)	b4401
Genitourinary and reproductive functions	Genital and reproductive functions	Sexual function	b640

Activity and Participation	**ICF Categories**	**Sub categories**	**ICF codes**
Learning and Applying knowledge	Applying knowledge	Reading	d166
		Writing	d170
	Purposeful sensory experience	Watching	d110
		Other purposeful sensory sensing e.g. appreciating pictures	d120
Communication	Communication- receiving	Communication with receiving spoken message	d310
		Communication with receiving nonverbal messages	d3150
		Communicating - producing	d345
Mobility	Changing and maintaining body position	Changing basic body position	d410
		Changing basic body position (Shifting the body's centre gravity)	d4106
	Carrying, moving and handling objects	Fine hand use (manipulating)	d4402
		Fine hand use (releasing)	d4403
		Hand and arm use (reaching)	d4452
	Walking and Moving	Walking	d450
		Walking on different surfaces	d4502
		Walking around obstacles	d4503
		Moving around in different locations (moving around outside the home and other buildings)	d4602
		Moving around using equipment	d465
	Moving around using transportation	Driving	d475
		Driving motorised vehicles	d4751
Self care	Caring for body parts		d520
		Caring for hair	d5202
		Caring for fingernails	d5203
		Caring for body parts, specified	d5208
Domestic life	Household tasks	Preparing meals	d6300
	Caring for household objects and assisting others	Making and repairing clothes	d6500
Interpersonal interactions and relationships	Complex interpersonal interactions		d720
	Particular interpersonal relationship	Informal social relationship	d750
Major life areas	Work and employment	Remunerative employment	d850
	Economic life	Basic economic transactions	d860
Community, social and civic life	Recreation and leisure	Play	d9200
		Sports	d9201
		Arts and culture	d9202
		Socialising	d9205
Environmental factors	**ICF Categories**	**Sub categories**	**ICF codes**
Natural environment and human-made changes to environment	Population	Population density	e2151
	Light	Light intensity	e2400
		Light quality	e2401
	Time related changes	Day /night cycles	e2450
Supports and relationship	Immediate family		e310
	Friends		e320
	People in positions of authority		e330
Services, systems & policies	Transportation services		e5400
